# Evaluation of anti-inflammatory and wound healing properties of *Tinospora cordifolia* extract

**DOI:** 10.1371/journal.pone.0317928

**Published:** 2025-01-29

**Authors:** Shohag Chandra Das, Subrato Biswas, Olin Khan, Rupa Akter, Md Abul Kalam Azad, Sujan Kumar Sarkar, Md. Abdul Masum, Sultana Bedoura

**Affiliations:** 1 Department of Dyes and Chemical Engineering, Bangladesh University of Textiles, Dhaka, Bangladesh; 2 Department of Anatomy, Histology & Physiology, Sher-e-Bangla Agricultural University, Dhaka, Bangladesh; 3 Department of Wet Process Engineering, Bangladesh University of Textiles, Dhaka, Bangladesh; Kerman University of Medical Sciences, ISLAMIC REPUBLIC OF IRAN

## Abstract

*Tinospora cordifolia* extract exhibits diverse benefits—anti-arthritis, anti-malarial, anti-allergic, anti-diabetic, antihepatotoxic, and antipyretic effects. Its specific anti-inflammatory and healing capacities remain unexplored, prompting a study utilizing a mouse skin wound model and direct *T*. *cordifolia* extraction. UV-Vis spectroscopy depicted an absorption range of 200–400 nm, while FTIR analysis identified alcohols, phenols, amines, amides, aldehydes, ketones, alkanes, and alkenes. GC-MS analysis revealed the presence of components: 5methyl-5-Hexen-2-Ol, n-hexadecenoic acid, cholesta-4,6-dien-3beta-ol, stigmasterol, β-sitosterol, stearate which are present in the extract. Histopathological examination confirmed accelerated wound healing, showcasing reduced inflammation, restored blood vessels, collagen fibers, and swift epidermal closure. *T*. *cordifolia* extract exhibits promise in enhancing wound healing through its antibacterial, anti-inflammatory properties.

## Introduction

Wound healing is an intricate biological process that is essential for tissue integrity renewal and functional restoration [1, 2]. It performs a crucial role in the body’s response when it is subjected to injuries whether they are caused by trauma, surgical procedure, or any underlying chemical, physical, or medical condition [3]. The process of wound healing is initiated immediately upon an injury and may persist for a different duration depending on the severity of the injury [4]. The whole procedure of wound healing consists of a multi-phased process that includes hemostasis, inflammation, wound contraction, re-epithelialization tissue, remodeling, and the formation of granulation tissue with angiogenesis [5, 6]. Even though the human body possesses a remarkable built-in mechanism for healing, it is not always flawless. Several factors can impact the wound healing process, such as patients’ health, size of the wound, bacterial infection, necrotic tissue, and blood supply interference [7]. These complications can lead to prolonged recovery time, increased medical costs, and harming the quality of life for the affected individuals if bacterial infection impedes wound healing [8]. As a result, combined anti-bacterial and anti-inflammatory treatment on the wound greatly speeds up the wound healing process [9].

In recent times, wound healing and antimicrobial treatment have been conducted by different antibiotics. Overuse or improper use of antibiotics or chemicals lead to the development of super bacteria, which exhibit resistance to most available antibiotic and medication [10]. In addition, the production cost of these antibiotics has skyrocketed recently and these man-made synthetics have certain side effects associated with them.

Lately, there has been a growing interest in using natural remedies in the field of medicine. Natural remedies have fewer adverse effects compared to synthetic pharmaceuticals, which makes them more suitable for long-term chronic conditions [11]. Moreover, unlike pharmaceutical drugs, natural remedies don’t require synthesis with extreme chemical synthesis, they can be sourced from plants and herbs which aligns with the sustainability goal.

In Southeast Asia, *Tinospora cordifolia*, belonging to the Menispermecea family, is commonly referred to by its traditional names, Amrita and Guduchi [12]. Its composition, comprising lignin, alkaloid, and terpenoid, has established its importance in Ayurvedic Medicine [13]. *T*. *cordifolia* extract has scientifically exhibited several therapeutic benefits, including serving as a general tonic, anti-inflammatory, anti-arthritis, anti-malarial, anti-allergic, anti-diabetic, antihepatotoxic and antipyretic [14, 15]. So, the continued scientific exploration of this plant has corroborated the long-standing beliefs advocated by traditional forms of medicine [12, 16, 17]. This plant species has been identified by the task force dedicated to the conservation and sustainable utilization of medicinal herbs as one of the most heavily exploited species in the pharmaceutical industry [18, 19].

Numerous prior studies have established wound healing capabilities of medicinal plants, including *Curcuma longa* [20], *Sphagneticola trilobate*, *Glycyrrhiza glabra*, *Kaempferia galanga*, *Apis mellifera* [21], *Mimosa tenuiflora* [18], *Camellia pubipetala* [22], *Spermadictyon suaveolens* [23], *Azadirachta indica* [24], *Sesamum indicum L*. [25] etc. among others. These plants possess a range of healing abilities, such as acute, chronic, incision, or closed wounds. There are some prior studies related to the application of pigment extracted from natural sources. A study was conducted to find out the efficacy of red pigment extracted from marine bacterium *Vibrio sp*. on wound healing and it showed a faster reduction in wound area compared to framycetin ointment-treated groups [26]. In another study by R. Poorniammal et al. pigments from methanolic extracts of *Thermomyces sp*. and *Penicillium purpurogenum* have wound healing and antimicrobial activities [9]. However, despite the acknowledged medicinal property of *Tinospora cordifolia*, any prior *in vivo* investigation into its potential for wound healing is notably lacking. Specially, with its direct extraction without any chemical application.

This study aims to analyze the wound healing property of *Tinospora cordifolia in vivo* condition on mice by histopathological analysis. The pigment has been extracted by a direct natural process to be used in vivo conditions and then centrifuged in a controlled manner to remove residual portions. Finally, the purified extract was used as a spray for applying puff spray. The name of the spray was given to Heal-Spray for the next commercial processes.

## Materials and method

### Materials

The entire herb, including stems with leaves, was methodically collected from Chandrima Uddan (Latitude 23.766533 and longitude: 90.378693), an open-access park in Dhaka, Bangladesh. Lush and vibrant herbs were selected and sharp scissors were employed to ensure minimal disturbance to neighboring plants. And they were labeled appropriate for storage and further analysis. The collection took place in the morning and to ensure the freshness and prevent the wilting or damage to the herb, they were secured and packaged in airtight plastic containers. Upon arriving at the Dyes and Chemical laboratory, the herbs were cleaned thoroughly to remove any dirt or debris. Then it was labeled and stored in the refrigerator at 4°C for further analysis.

### Extraction of plant pigment and spray preparation

**Fig 1:**
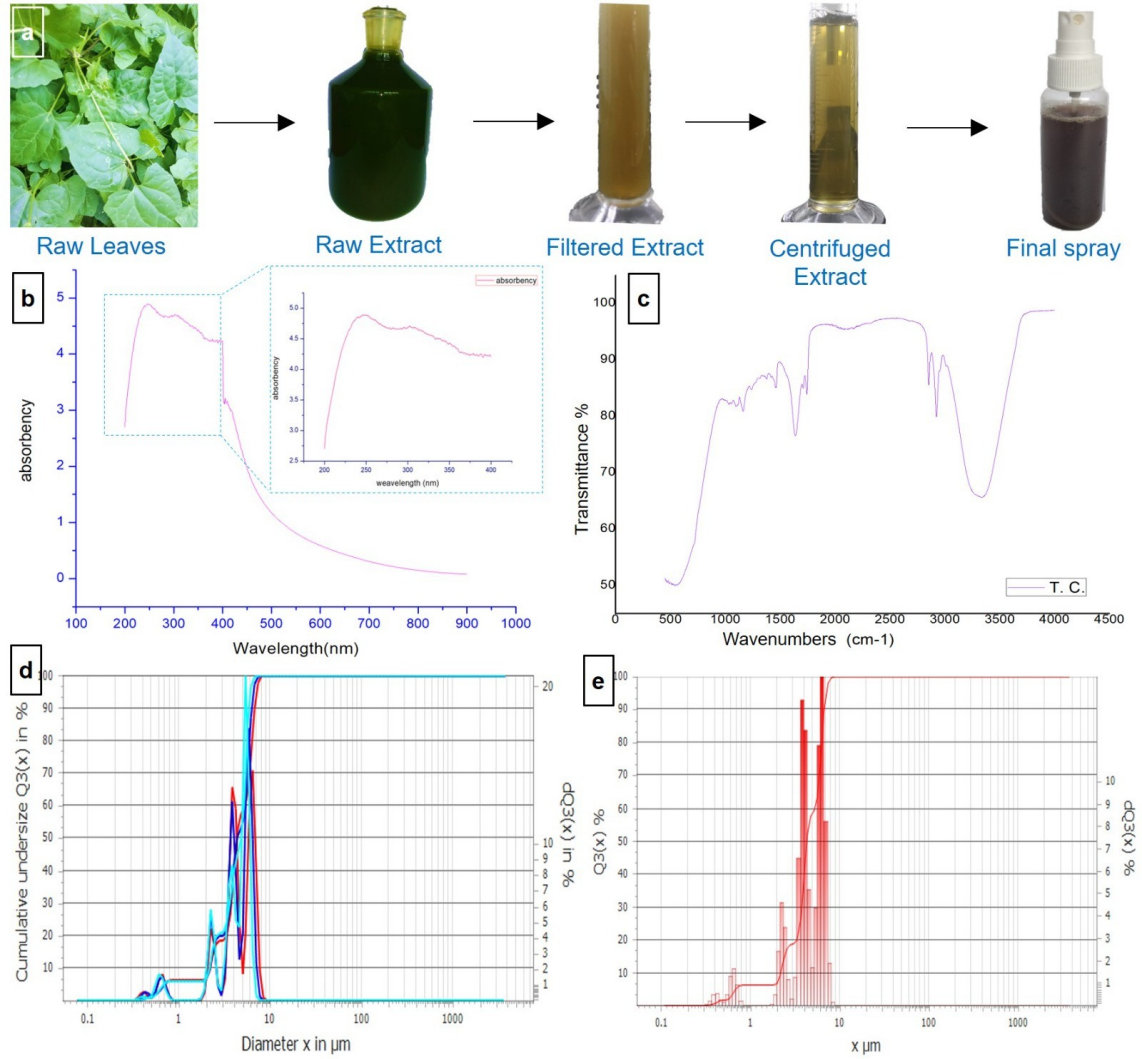
Extraction and physiochemical characterization of the extracted pigment. (**a**) Heal-Spray production from the plant of *T*. *cordifolia* extract by direct extraction method; (**b**) UV-Vis spectroscopy of *T*. *cordifolia* extract after centrifuging showing better absorbency in the ultraviolet region;(**c**) Fourier transform infrared (FTIR) spectra of *T*. *cordifolia*; (**d** & **e**) Particle size of pigments in *T*. *cordifolia* extract.

A manual metal crusher was employed to extract the pigment from the plant. All the stored herbs were transferred into metal crushed and it was crushed directly to extract the liquid from the plant. The liquid was kept in a glass container for precipitation of debris particles and then filtered. After that, a centrifuging machine (3000 rpm -10 min) was used to separate fine solids from the solution. Then the extracted pure solution was transferred into a 100 ml spray bottle for application and was stored in the refrigerator at 4°C for further analysis **Fig 1A**.

### Physicochemical characterization

The UV−visible absorption spectroscopy of the plant pigment extract was conducted by PerkinElmer UV/VIS Lambda 365 (PerkinElmer, Inc., USA). Fourier transform infrared (FTIR) spectra were recorded on Spectrum two FTIR spectrometers, Perkin Elmer. The particle size of the pigments was detected by FRITSCH ANALYSETTE 22 NeXT (FRITSCH Asia-Pacific Pte. Ltd, Singapore). The Gas Chromatography-Mass spectrometry (GC-MS) analysis was conducted by a similar procedure with a similar machine (GCMS-QP2010 SE, Japan) [27].

### Antibacterial assay

The antibacterial potential of extracted pigment was assessed at Waffen Research Laboratory, Dhaka. The agar diffusion method was followed to conduct an antibacterial test. Two strains of bacteria (*Streptococcus aureus*: ATCC 6538 as Gram-positive and *Escherichia coli*: ATCC 11775 as Gram-negative) were used. Wells were made in the agar to which plan extract was added. The agar plates were then incubated for 24 hours to allow the growth of the bacteria. The bacterial cell concentration was 1000 CFU/ml. After incubation, the plates were examined for zones of inhibition, which appear as clear areas around the wells where microbial growth had been inhibited by the test substance. The mean diameter of the zone of inhibition (ZOI) around the disc in millimeters (mm) was measured and compared.

### *In vivo* assay

#### Ethical statement

The Approval Committee for Animal Experiments, Faculty of Animal Science and Veterinary Medicine, Sher-e-Bangla Agricultural University, approved the study (Memo no: SAU/AHiPhy/22/843). The animals (albino mice) were fed a regular pellet diet and provided tap water. Throughout the study, all animals were given human care by the requirements specified in the "Guide for the Care and Use of Laboratory Animals."

#### Experimentally induced excision wounds

Prior to creating the wounds, the animals were sedated with ketamine (80 mg/kg body weight) and xylazine (15 mg/kg body weight). Hair on the skin was removed using an electric clipper, cleansed with 70% alcohol, and injected with 1 ml of Lignocaine HCl (2%, 100 mg/5 ml). With the use of a round seal, an area of uniform wound 4.5 mm in diameter was excised from the back of all mice. During the surgery, no incisions were made in the muscle layer, and skin tension was kept constant. The wound was completely open. The wound area was promptly measured by drawing it with transparent tracing paper over the wound. The left wound was retained as the control, which received a vehicle whereas the right wound was the treatment, which received the 1 puff spray per time, 3 times a day. Each animal’s wound closure area was measured by tracing the wound on days 5, 10, and 15 after wounding surgery, and the wound closure rate was presented in millimeters.

#### Histological evaluation of wounds

After complete wound healing, five mice from each time period were euthanized with an overdose of ketamine and xylazine. Skin samples from the wound sites were fixed in 10% neutral buffered formalin (NBF) and subsequently processed using a paraffin tissue processing machine. A 3μm segment of the wound was then stained with hematoxylin and eosin (H&E) for skin histopathology analysis.

#### Statistical analysis

Statistical analyses were conducted to evaluate the effectiveness of the treatment on wound closure over different time periods. A repeated measures ANOVA was performed using Minitab (Version 18.0) to determine if there were statistically significant differences in wound closure diameters at 0 days, 5 days, 10 days, and 15 days within both the control and treatment groups. Post hoc comparisons were made using Tukey’s Honest Significant Difference (HSD) test to identify specific time points where significant differences occurred. Additionally, the Least Significance Difference (LSD) test was utilized via Python (Version 3.11.5) to compare wound closure diameters between the control and treatment groups at each time point. Statistical significance was set at p < 0.05, and all results are presented as mean ± standard deviation (SD). Detailed statistical results are provided in the supplementary tables (S1A-S1F Tables in S1 File).

## Results and discussions

### Physicochemical characteristics

As *T*. *cordifolia* is a medicinal plant, the direct extract was converted into puff spray for easy care and application. The UV-Vis spectroscopy in **Fig 1B** revealed absorption within the ultraviolet spectral range of 200 to 400 nm, with the peak absorbance occurring at 250 nm. This specific absorbance pattern suggests the presence of secondary metabolites in the extract of *T*. *cordifolia*. Secondary metabolites containing π-bonds, σ- σ-bonds, chromophores, and aromatic rings with lone pairs of electrons were confirmed by the spectra [28]. Among the two types of metabolites present in the cells of *T*. *cordifolia*, the secondary metabolites are the derived products of primary metabolites like proteins, lipids, carbohydrates, crude fiber, vitamins, and fats. The secondary metabolites are flavonoids, terpenoids, lignins, alkaloids, Phenol, tannins, sterols, etc. involved in the metabolic activity [28]. Some of the metabolisms present in *T*. *cordifolia* are anticancer, antimalarial, anti-diuretic, antibacterial activities, anti-viral, anti-analgesic, antidiabetic activities, etc [29] which also concurrents with several related studies [30–32].

The presence of these secondary metabolites (alcohol, alkanes, phenol, alkenes, amine compound, aromatic, aliphatic amines, etc.) is also confirmed by FTIR spectra **Fig 1C**. The broad peak at 3340 cm^-1^ indicates the presence of O-H stretch and H bond which could confirm the presence of alcohols and phenols. The sharp peak at 2923 cm^-1^ indicates the presence of (C-H) aromatic ring and at 2853 cm^-1^ associates with primary, secondary amines and amides (NH).

Again, a small sharp peak at 1743 cm^-1^ confirms the presence of aldehydes and ketones (C = O) and C-H stretch at 1637 cm^-1^ as alkanes. Another small peak at 1463 cm^-1^ indicates -C = C- stretch for alkenes. The peak at 1239 cm^-1^ indicates the presence of alkyl halides (C-H). All of these spectra data merge with previous studies of *T*. *cordifolia* extract with different methods [28, 33]. The data suggests that the direct extraction of the pigment did not hamper the metabolite compounds.

**Fig 1D** and **1E** shows the size of the pigments present in the centrifuged extract. The sizes range from 0.5 μm to 10 μm, highlighting the extract’s versatility as a pigment for various applications. The particle size requirements vary based on intended use, ranging from fine (<10 μm) to coarse (>45 μm) grades [34].

Among the 68 components detected in GC-MS in **Table 1**, major 24 components were tabulated with 92.50%. The chemicals present in *T*. *cordifolia* contain different pharmacological importance. Different components have different purposes, including antioxidant activity, antimicrobial activity, anti-toxic effects, antidiabetic activity, antistress activity, hypolipidemic effect, hepatic disorder, anticancer activity, anti-HIV potential, wound healing, anti-osteoporotic effects, anticomplement activity, and immunomodulating activity, etc. [29]. The main component was n-hexadecenoic acid with 22.37% which is an anti-inflammatory and antioxidant agent [35]. The second highest component was octadecanoic acid with 9.9 6% which is an antimicrobial agent [36].

**Table 1:** Gas Chromatography-Mass spectroscopy (GC-MS) of the plant extract of *T*. *cordifolia* (major components with amounts more than 1% have been listed among 68 components).

SI No	Compounds	Chemical Group	Molecular Formula	Retention time (min)	Area%
1	4-methyl-1,3-dioxane	Ether	C_5_H_10_O_2_	4.51	1.11
2	3,5-dihydroxy-6-methyl-2,3-dihydropyran-4-one	Ketone	C_6_H_8_O_4_	5.35	2.74
3	5 methyl-5-Hexen-2-Ol	Alcohol	C_7_H_14_O	7.95	1.03
4	2-pentylcyclopentan-1-one	Ketone	C_10_H_18_ O	9.09	4.22
5	1-hexadecene	Alkene	C_16_H_32_	11.52	1.19
6	8-pentadecaanone	Ketone	C_15_H_30_O	12.47	3.05
7	9-octadecenoic acid	Acid	C_18_H_34_O_2_	13.79	1.98
8	6,10,14-trimethylpentadecan-2-one	Ketone	C_18_H_36_O	14.87	3.34
9	8-octadecanone	Ketone	C_18_H_36_O	15.02	1.51
10	7-hexadecenoic acid methyl ester	Ester	C_17_H_32_O_2_	15.23	1.64
11	N-hexadecenoic acid	Acid	C_16_H_32_O_2_	15.87	22.37
12	Octadecanoic acid	Acid	C_17_H_34_O_2_	17.69	9.96
13	Ethyl heptadecanoate	Ester	C_19_H_38_O_2_	18.12	1.03
14	8,11,14-Eicostrienoic acid	Acid	C_20_H_34_O_2_	18.80	1.62
15	Icosanoic acid	Acid	C_15_H_22_O	19.65	2.28
16	Andrographolide	Terpenoid	C_20_H_30_O_5_	21.11	1.38
17	1-heptacosanol	Alcohol	C_27_H_56_O	24.57	3.17
18	Cholesta-4,6-dien-3beta-ol	Alcohol	C_27_H_44_O	27.13	6.01
19	(24R)-Methylcholest-5-en-3beta-ol	Alcohol	C_28_H_48_O	29.38	1.70
20	Stigmasterol	Alcohol	C_29_H_48_O	29.99	3.27
21	β-sitosterol stearate	Acid salt	C_47_H_84_O_2_	31.92	4.55
22	24-methylenecycloartanol	Alcohol	C_31_H_52_O	32.01	1.76
23	Stigmasta-4,22-dien-3-one	Ketone	C_29_H_46_O	33.92	2.03
24	Cholest-4-en-3-one	Ketone	C_27_H_44_O	35.33	9.56
				**Total**	**92.50**

However, for being a potential wound healing agent, the components that work as the initiator of faster healing as an anti-inflammatory agent are present in the pigment. Here, 5 methyl-5-hexen-2-Ol, n-hexadecenoic acid, 9-octadecenoic acid, Cholesta-4,6-dien-3beta-ol, stigmasterol, β-sitosterol stearate and cholest-4-en-3-one are present respectively as 1.03%, 1.98%, 22.37%, 1.98%, 6.01%, 3.27%, 4.55% and 9.56%. These components are potential anti-inflammatory agents [15, 37]. 9-octadecenoic acid restricts the nitric oxide (NO) production during inflammation for optimum RAW264.7 macrophages induced by lipopolysaccharide (LPS) [38]. Structural analysis and kinetic study showed the anti-inflammatory properties of n-hexadecenoic Acid [39]. Cholesta-4,6-dien-3beta-ol is a derivative of cholestane (C431700) and cholesterol (C432501) that initiate the cell signaling for the wound healing process [40]. Stigmasterol is a tetracyclic triterpene which is a common plant sterol. It is an unsaturated phytosterol that has already shown anti-inflammatory actions against several metabolic disorders both in vitro and in vivo conditions [41]. Moreover, 4-methyl-1,3-dioxane can be transferred to n-[2-(5,5-dimethyl-1,3-dioxane-2-yl) ethyl] amino acid which is an anti-inflammatory drug [42].

All the physiochemical analysis proves the suitability of the direct *T*. *cordifolia* extract as plant pigment for the next process. However, the outcome of the GC-MS analysis does not rule out the possibility of crosschecking the components with standards, which can be done in future studies to confirm the findings.

### Antibacterial property

The antibacterial property of pigment extract is crucial for wound healing because it hinders the growth of harmful microorganisms, preventing any infections caused by those pathogens. This not only inhibits the pathogen proliferation but also accelerates wound healing.

**Fig 2:**
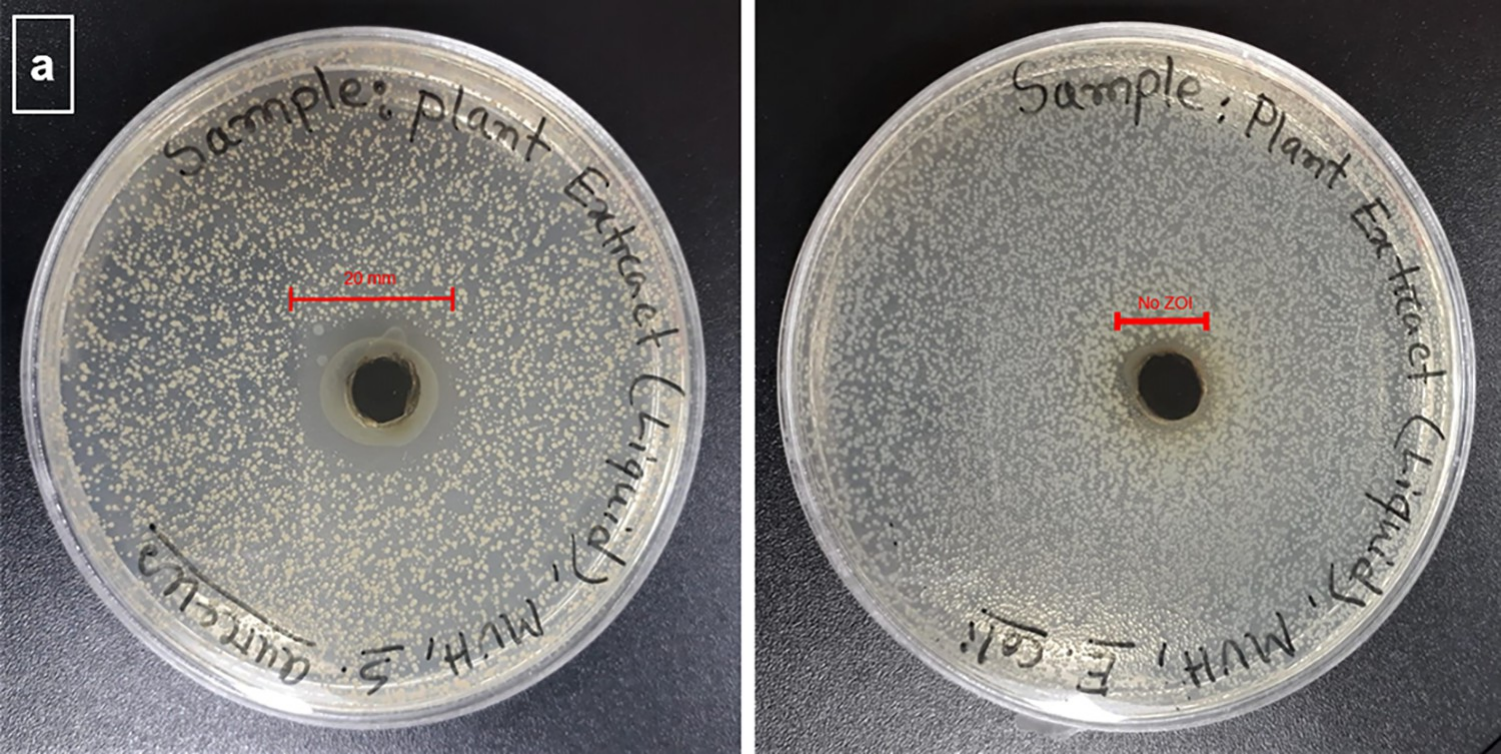
Zone of Inhibition (ZOI) of *T*. *Cordifolia* extract against Gram-positive and Gram-negative bacteria (Streptococcus aureus: ATCC 6538-Left and Escherichia coli: ATCC 11775-Right).

**Fig 2** displays the zone of inhibition (ZOI) values for the pigment extract against both bacterial strains. Overall, the pigment exhibited antibacterial Gram-positive bacteria but was not able to show any activity against Gram-negative bacteria. The pigment had 20 mm ZOI against Gram-positive *E*. *coli*, which proved that *T*. *cordifolia* is capable of killing Gram bacteria. However, no ZOI was seen on the inoculated plate for Gram-negative bacteria. This antibacterial property could be attributed to the fact the pigment contains bioactive compounds such as alkaloids and glycosides, which disrupt the microbial cell membrane and disrupt the metabolic process, ultimately leading to the killing or inhibiting the proliferation of the pathogens. From GC-MS, Octadecanoic acid, 1-hexadecene, and 8-pentadecaanone are possible components for antibacterial properties [36, 43].

### Histopathology assay

#### Acute toxicity

Clinical observations, blood biochemistry, hematology, and histopathology data did not reveal any appreciable differences between the control and treated groups after the animals were given the *T*. *cordifolia* leaf extract at doses of 1, 2, and 5 g/kg and monitored for 14 days. All the mice survived and did not manifest any significant visible toxicity at these doses.

#### Gross wound closure in control and treatment group

**Fig 3:**
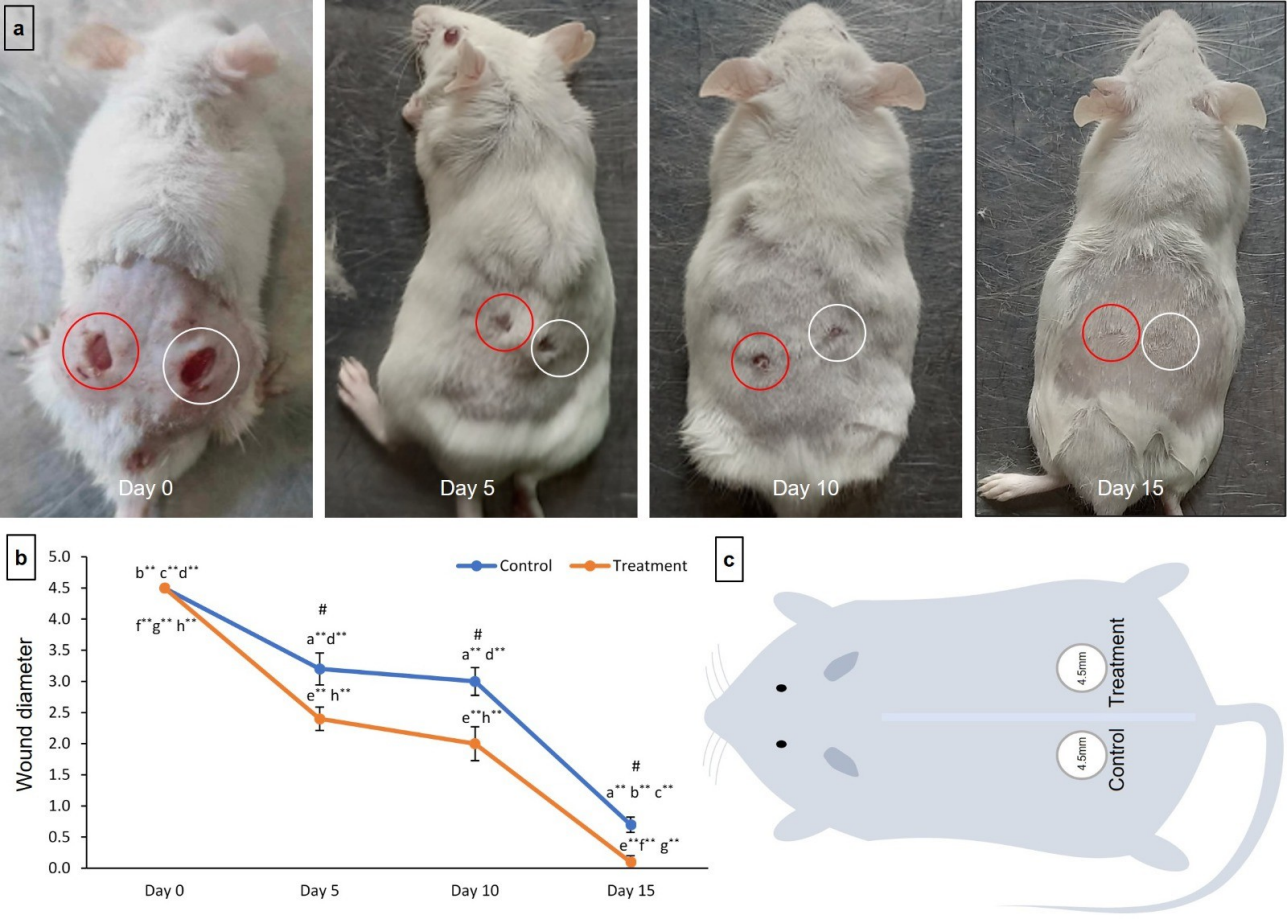
Gross wound closure analysis. (**a**) Gross wound closure in the control group (red circle) and treatment group (white circle) at 4 different periods (day 0, day 5, day 10, and day 15); (**b**) (**b**) Measurement of average wound closure diameter for each group (N = 5) at different time periods (at 0, 5, 10, and 15 days). * and ** indicate significance within a group (P<0.05 and P<0.01) and ^#^indicates significance between control and treatment group at different time periods (P<0.05). a, b, c, d, e, f, g and h denote wound closure at 0-day control, 5-day control, 10-day control, 15-day control, 0-day treatment, 5-day treatment, 10-day treatment, and 15-day treatment, respectively. Repeated ANOVA test followed by Post Hoc Tukey Test and Least Significance Difference test; (**c**) Work design of the Process of wound healing *in vivo* assay on mice.

In comparison to the control group, the treatment groups exhibited significantly accelerated wound closure (**Fig 3A**). **Fig 3B** and **3C** depict the wound closure progress at 5, 10, and 15 post-wounding surgeries for both the control and treatment groups, as well as the experimental design for the in vivo wound healing assay conducted on mice.

To evaluate the differences in wound closure diameters over time, a repeated measures ANOVA was conducted across four time points: 0 days, 5 days, 10 days, and 15 days. The analysis revealed significant differences in wound diameters within both the control and treatment groups. For the control group, the ANOVA yielded an F-value of 105.1228 with a p-value less than 0.0001, indicating a highly significant result. Similarly, the treatment group showed an F-value of 110.0845 with a p-value less than 0.0001, also indicating high statistical significance (see Tables S1A-S1C in S1 File for detailed results).

Post hoc comparisons using the Tukey test further dissected these differences. In the control group, significant differences were observed between day 0 and all subsequent days, as well as between day 5 vs. day 15 and day 10 vs. day 15. No significant difference was found between day 5 and day 10 (S1D Table in S1 File). The treatment group exhibited a similar pattern, with significant differences between day 0 and all subsequent days, and between day 5 vs. day 15 and day 10 vs. day 15, but no significant difference between day 5 and day 10 (S1E Table in S1 File).

Additionally, the Least Significant Difference (LSD) test was used to compare wound closure diameters between the control and treatment groups at each time point. The results indicated significant differences at 5 days, 10 days, and 15 days, but no significant difference at 0 days (S1F Table in S1 File).

These findings underscore the effectiveness of the treatment over time, demonstrating its impact on accelerating wound closure compared to the control.

#### Histopathology

**Fig 4:**
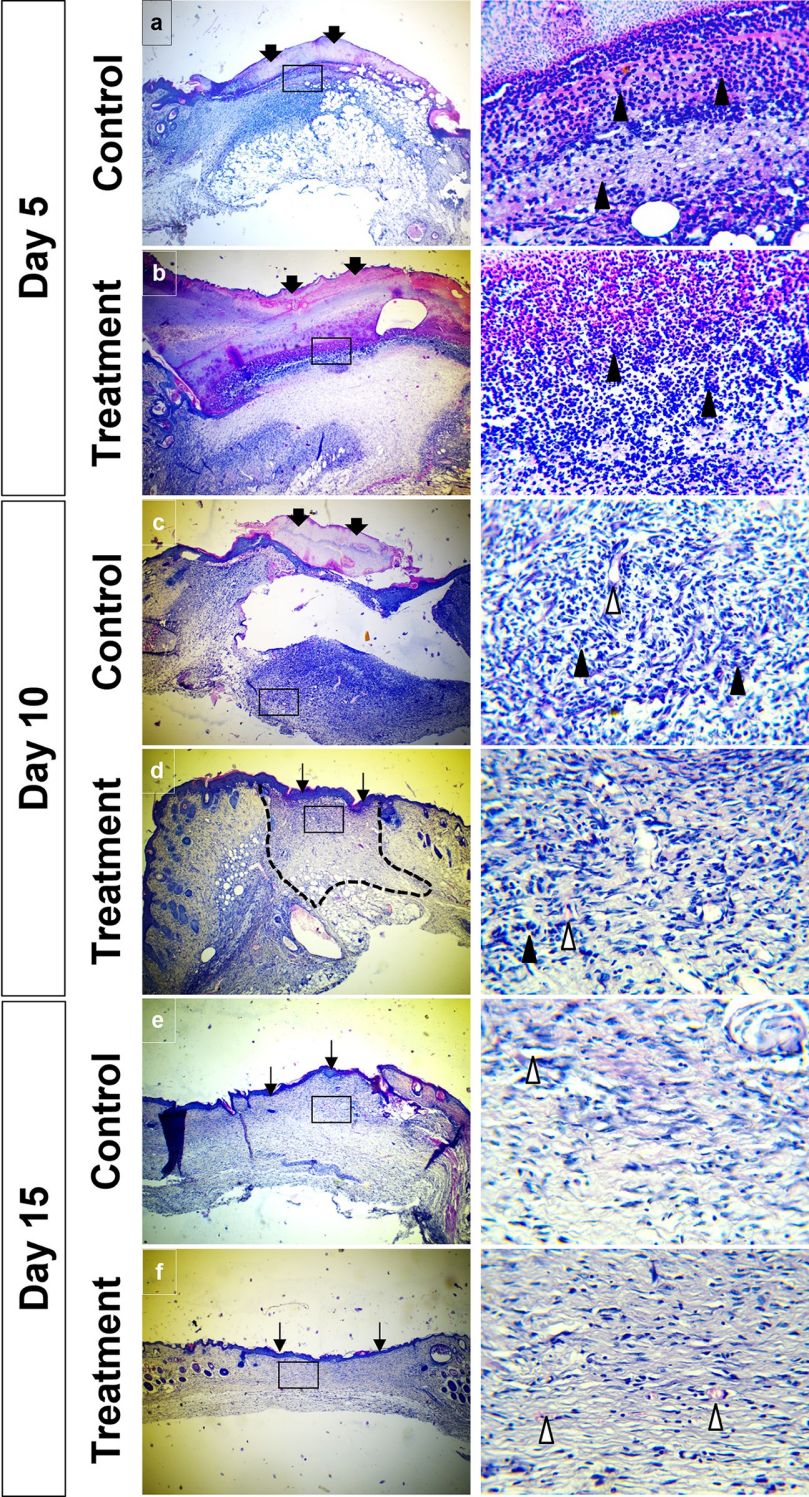
Wound healing at different periods. The vehicle control group shows a thin scab (thick arrow) on the top of the wound and infiltrating cells (arrowhead, inset) in the wound (**a**) on day 5. The treatment group shows a thickened scab on top of the wound and infiltrating cells (arrowhead, inset) in the wound (**b**) on day 5. The vehicle control group still shows a scab on the top of the wound and infiltrating cells (arrowhead, inset) in the wound (**c**) on day 10. The treatment group shows the disappearance of the scab but the formation of scar tissue (dashed area) on the wound with very few infiltrating cells in the wound (arrow, inset) and the appearance of a newly formed epidermis (arrow) (**d**) at day 10. In treatment groups, blood vessels (empty arrowhead) are more typical in form. The vehicle control group shows the appearance of a newly formed epidermis (arrow) and few collagen fibers in the dermal area (**e**) on day 10. Blood vessels (empty arrowheads) are not in typical form. The treatment group shows more collagen fiber and typical blood vessels (empty arrowhead) in the dermal area of the treatment group (**f**) at 15 days.

On day 5, the vehicle control group exhibited a thin scab atop the wound and infiltrating cells in the wound (**Fig 4A)**, whereas the treatment group displayed a thicker scab and infiltrating cells (**Fig 4B)**. By day 10, the vehicle control group still had a scab and infiltrating cells in the wound (**Fig 4C)** while the treatment group showed scab disappearance, scar tissue formation, few infiltrating cells in the wound and newly formed epidermis (**Fig 4D)**. The treatment group also exhibited more typical blood vessels formation. On day 10, the vehicle control group displayed newly formed epidermis and few collagen fibers in the dermal area (**Fig 4E)** with atypical blood vessels. In contrast, the treatment group exhibited more collagen fibers and typical blood vessels in the dermal area by day 15 (**Fig 4F)**.

Topical wound medications, including sprays, ointments, and bandages, are commonly used for surface wounds due to their direct delivery to the affected area. It is noteworthy to highlight that the extract did not irritate or hurt mice over the entire duration of wound therapy since they did not exhibit any symptoms of restlessness or scratching or biting at the wound site while the extract was administered. It is consistent with earlier research, which showed that the extract had no toxic effects [4]. Wound healing is the body’s natural response to injuries, characterized by a dynamic and intricate process that enable the rapid restoration of healthy tissues and function [44]. The initial and shortest stage of wound healing is hemostasis which involves processes that stop bleeding. At the cut end of a constricted conduit, platelets quickly cluster together, cling to the connective tissue, and form a fibrin clot when the clotting cascade is activated [45, 46]. In this study, thicker scabs observed in the treatment group compared to the control group are indicative of the treatment group’s initiation of a healthy healing process.

Following homeostasis, tissue damage accompanied by the production of cytokines and other inflammatory mediators results in the recruitment of neutrophils as a second responding cell to the site of the wound. These cells perform bacterial lysis via oxidative burst processes, debris scavenging, complement-mediated opsonization, and engulfment and lysis of foreign organisms [45, 47]. Additionally, macrophages trigger EGF and PDGF, which induce the development of granulation tissue and the shift from the proliferative phase to tissue regeneration [4].

Inflammation starts immediately after a wound occurs and typically lasts for one to four days. While this stage is crucial for the healing process, prolonged inflammation can potentially impede regeneration [48]. In this study, scar tissue appeared earlier in the treatment group compared to the control group. Additionally, the treatment group showed a rapid reduction in mononuclear cells. During the proliferative stage of healing, the wound site undergoes several interconnected processes, comprising re-epithelialization, angiogenesis, collagen formation, and extracellular matrix (ECM) production, to rebuild the vascular network and form granulation tissue [44]. The current study revealed early re-epithelialization, the formation of normal blood vessels, and a greater buildup of collagen fibers in the treatment group, all of which indicate effective and faster healing facilitated by *Tinospora cordifolia* extract.

In conclusion, the pigment extract from this plant has exhibited remarkable wound-healing properties, suggesting its potential for treating a wide range of wounds in humans and domestic animals.

## Conclusion

The direct extract from *Tinospora cordifolia* is a viable source of medicinal application as a puff spray. It is clear from the physiochemical analysis that the direct extract showed comparable physiochemical properties to the methanolic extracts of previous studies [49, 50]. The absorption range within 200nm - 400nm determined by UV-Vis spectroscopy and FTIR peaks at 3340 cm^-1^, 2923 cm^-1^, 2853 cm^-1^, 1743 cm^-1^, 1637 cm^-1^, 1239 cm^-1^ indicated the presence of secondary metabolites in the extract. GC-MS analysis corroborated this finding. Histopathological analysis on day 5 post-application of *T*. *cordifolia* extract showed a thicker scab and increased infiltrating cells in the treatment group compared to the control. By day 10, the treatment group exhibited scar tissue, reduced infiltrating cells, and newly formed epidermis, along with more typical blood vessel formation, contrasting with the control group’s scab, few collagen fibers in the dermal area, and atypical blood vessels.

The extract from *T*. *cordifolia* promotes early re-epithelialization, formation of typical blood vessels, and increased deposition of collagen fibers, leading to effective and faster healing. The positive effect of Heal-Spray on acute wounds in mice suggests its potential for commercialization. Specifically, the treated wounds exhibited superior histological outcomes compared to the control, indicating a healthy healing process. Therefore, it can be inferred that Heal-Spray is a natural pharmaceutical with the potential to aid in wound healing.

## Supporting information

S1 File Statistical analysis. ANOVA analysis for wound closure.
(PDF)

## References

[1] Mathew-steiner SS, Roy S, Sen CK. Collagen in Wound Healing. 2021; 10.3390/bioengineering8050063 PMC8151502 34064689

[2] Gurtner GC, Werner S, Barrandon Y, Longaker MT. Wound repair and regeneration. 2008;453(May). 10.1038/nature07039 18480812

[3] Sorg H, Tilkorn DJ, Mirastschijski U, Hager S. Skin Wound Healing: An Update on the Current Knowledge and Concepts. 2017;81–94. 10.1159/000454919 27974711

[4] Al-henhena NA, Fanous S. Histological study of wound healing potential by ethanol leaf extract of Strobilanthes crispus in rats. 2011;(August).

[5] Attinger CE. Wound Healing: An Overview. 2006;1–32. 10.1097/01.prs.0000222562.60260.f9 16801750

[6] Eming SA, Martin P, Tomic-canic M. STATE OF THE ART REVIEW Wound repair and regeneration: Mechanisms, signaling, and translation. 2014;6(265). 10.1126/scitranslmed.3009337 PMC4973620 25473038

[7] Anderson K, Hamm RL. Factors That Impair Wound Healing. J Am Coll Clin Wound Spec [Internet]. 2014;4(4):84–91. Available from: doi: 10.1016/j.jccw.2014.03.001 26199879 PMC4495737

[8] Zhao H, Huang J, Li Y, Lv X, Zhou H, Wang H, et al. Biomaterials ROS-scavenging hydrogel to promote healing of bacteria infected diabetic wounds. Biomaterials [Internet]. 2020;258(August):120286. Available from: 10.1016/j.biomaterials.2020.120286 32798744

[9] Poorniammal R. Antimicrobial and wound healing potential of fungal pigments from Thermomyces sp. and Penicillium purpurogenum in wistar rats Antimicrobial and wound healing potential of fungal pigments from Thermomyces sp. and Penicillium purpurogenum in wistar rats. 2022;(July).

[10] Miao J, Pangule RC, Paskaleva EE, Hwang EE, Kane RS, Linhardt RJ, et al. Lysostaphin-functionalized cellulose fibers with antistaphylococcal activity for wound healing applications. Biomaterials. 2011;32(36):9557–67. doi: 10.1016/j.biomaterials.2011.08.080 21959009

[11] Modi B, Shah KK, Shrestha J, Shrestha P, Basnet A, Tiwari I, et al. Advanced Journal of Chemistry-Section B Natural Products and Medical Chemistry Morphology, Biological Activity, Chemical Composition, and Medicinal Value of Tinospora Cordifolia (willd.) Miers. 2020;(October). Available from: http://www.ajchem-b.com/

[12] Basalingappa KM. Tinospora cordifolia: The Antimicrobial Property of the Leaves of Amruthaballi. J Bacteriol Mycol Open Access. 2017;5(5):363–71.

[13] Shukla A, Rasik AM, Jain GK, Shankar R, Kulshrestha DK. In vitro and in vivo wound healing activity of asiaticoside isolated from Centella asiatica. 1999;65:1–11. 10.1016/s0378-8741(98)00141-x 10350364

[14] Naik D, Dandge C, Rupanar S. Determination of Chemical Composition and Evaluation of Antioxidant Activity of Essential Oil from Tinospora cordifolia (Willd.) Leaf. J Essent Oil-Bearing Plants. 2014;17(2):228–36.

[15] Sharma U, Bala M, Kumar N, Singh B, Munshi RK. Immunomodulatory active compounds from Tinospora cordifolia. J Ethnopharmacol [Internet]. 2012;141(3):918–26. Available from: doi: 10.1016/j.jep.2012.03.027 22472109

[16] Das SC, Uddin MA. Extraction and Characterization of Antimicrobial surgical Suture from the bast of Tinospora Cordifolia Extraction and Characterization of Antimicrobial surgical Suture from the bast of Tinospora Cordifolia. J Nat Fibers [Internet]. 2022;00(00):1–11. Available from: 10.1080/15440478.2021.2022560

[17] Singh D, Chaudhuri PK. Chemistry and pharmacology of Tinospora cordifolia. Nat Prod Commun. 2017;12(2):299–308. 30428235

[18] Kumarasamyraja D, Jeganathan NS, Manavalan R. A Review on Medicinal Plants with Potential Wound Healing Activity. 2012;2(4):101–7.

[19] Khanal P, Patil BM, Mandar BK, Dey YN, Duyu T. Network pharmacology-based assessment to elucidate the molecular mechanism of anti-diabetic action of Tinospora cordifolia. Clin Phytoscience. 2019;5(1):1–9.

[20] Rev P, Jain S, Shrivastava S, Nayak S, Sumbhate S. PHCOG MAG.: Plant Review Recent trends in Curcuma Longa Linn. 2007;1(1):119–28.

[21] Sharma A, Khanna S, Kaur G, Singh I. Medicinal plants and their components for wound healing applications. 2021;

[22] Yang CS, Chen G, Wu Q. Recent Scientific Studies of a Traditional Chinese Medicine, Tea, on Prevention of Chronic Diseases. J Tradit Complement Med [Internet]. 2014;4(1):17–23. Available from: doi: 10.4103/2225-4110.124326 24872929 PMC4032838

[23] Rani S, Shalu R, Prakash GS, Kapil K, Sukhbir K. REVIEW ARTICLE WOUND HEALING POTENTIAL OF MEDICINAL PLANTS WITH THEIR SCREENING MODELS: A COMPREHENSIVE REVIEW Inflammation Proliferation and Reconstruction Maturation and Remodeling. 2016;6(1):56–66.

[24] Phd-corresponding OEA, Mbbs ATA. The Wound Healing Effects of Aqueous Leave Extracts of Azadirachta Indica on Wistar Rats. 2013;3(6):181–6.

[25] Kiran K, Asad M. Wound healing activity of Sesamum indicum L seed and oil in rats. 2008;46(November):777–82. 19090349

[26] Nayak BS, Isitor GN, Maxwell A, Bhogadi V, Ramdath DD. Wound-healing activity of.: 83–6. 10.12968/jowc.2007.16.2.27006 17319624

[27] Chandra Das S, Hossain M, Hossain MZ, Jahan N, Uddin MA. Chemical analysis of essential oil extracted from pomelo sourced from Bangladesh. Heliyon [Internet]. 2022;8(12):e11843. Available from: doi: 10.1016/j.heliyon.2022.e11843 36478837 PMC9720520

[28] Papitha R, Lokesh R, Kaviyarasi R, Selvaraj CI. Phytochemical screening, FT-IR and gas chromatography mass spectrometry analysis of Tinospora cordifolia (Thunb.) Miers. Int J Pharmacogn Phytochem Res. 2016;8(12):2020–4.

[29] Sharma P, Dwivedee BP, Bisht D, Dash AK, Kumar D. The chemical constituents and diverse pharmacological importance of Tinospora cordifolia. Heliyon [Internet]. 2019;5(9):e02437. Available from: doi: 10.1016/j.heliyon.2019.e02437 31701036 PMC6827274

[30] Surya M, Sampath S, Vairamuthu SB, Sravanthy PG, Ramachandran B, Al-Ansari MM, et al. Aloe vera—mediated silver-selenium doped fucoidan nanocomposites synthesis and their multi-faceted biological evaluation of antimicrobial, antioxidant and cytotoxicity activity. Mater Technol [Internet]. 2024;39(1). Available from: 10.1080/10667857.2024.2331899

[31] Aaditya Marthandan P, Geetha Sravanthy P, Snega R, Carmelin D, Surya M, Catakapatri Venugopal D, et al. Melothria maderaspatana mediated one-pot synthesis of cerium-doped Silymarin nanoparticles and their antibacterial and anticancer studies. Mater Technol [Internet]. 2024;39(1). Available from: 10.1080/10667857.2024.2315381

[32] Mathesh A, Carmelin DS, Mohanprasanth A, Geetha Sravanthy P, Snega R, Surya M, et al. Tridax procumbens–mediated one pot synthesis of silver-doped fucoidan nanoparticles and their antibacterial, antioxidant, and anti-inflammatory efficacy. Biomass Convers Biorefinery. 2024;14(8):9887–96.

[33] Ramachandra YL, A KHB. “F TIR, Uv- Vis And GCMS Analysis Of Potential Bioactive Compounds From Tinospora Cordifolia And Its Antibacterial Activity.” 2023;10:3738–48.

[34] Sekar N. Optical effect pigments for technical textile applications. Adv Dye Finish Tech Text. 2013;37–46.

[35] Siswadi S, Saragih GS. Phytochemical analysis of bioactive compounds in ethanolic extract of Sterculia quadrifida R.Br. AIP Conf Proc. 2021;2353(September 2018).

[36] hui Pu Z, qun Zhang Y, qiong Yin Z, Xu J, yong Jia R, Lu Y, et al. Antibacterial activity of 9-octadecanoic acid-hexadecanoic acid-tetrahydrofuran-3,4-diyl ester from neem oil. Agric Sci China [Internet]. 2010;9(8):1236–40. Available from: 10.1016/S1671-2927(09)60212-1

[37] Paniagua-Pérez R, Flores-Mondragón G, Reyes-Legorreta C, Herrera-López B, Cervantes-Hernández I, Madrigal-Santillán O, et al. Evaluation of the Anti-Inflammatory Capacity of Beta-Sitosterol in Rodent Assays. African J Tradit Complement Altern Med AJTCAM. 2017;14(1):123–30. doi: 10.21010/ajtcam.v14i1.13 28480389 PMC5411862

[38] Xie C, Wang S, Cao M, Xiong W, Wu L. (E)-9-Octadecenoic Acid Ethyl Ester Derived from Lotus Seedpod Ameliorates Inflammatory Responses by Regulating MAPKs and NF- B Signalling Pathways in LPS-Induced RAW264.7 Macrophages. Evidence-based Complement Altern Med. 2022;2022. 10.1155/2022/6731360 PMC8754602 35035506

[39] Aparna V, Dileep K V., Mandal PK, Karthe P, Sadasivan C, Haridas M. Anti-Inflammatory Property of n-Hexadecanoic Acid: Structural Evidence and Kinetic Assessment. Chem Biol Drug Des. 2012;80(3):434–9. doi: 10.1111/j.1747-0285.2012.01418.x 22642495

[40] Larouche J, Sheoran S, Maruyama K, Martino MM. Immune regulation of skin wound healing: Mechanisms and novel therapeutic targets. Adv Wound Care. 2018;7(7):209–31. doi: 10.1089/wound.2017.0761 29984112 PMC6032665

[41] Bakrim S, Benkhaira N, Bourais I, Benali T, Lee LH, El Omari N, et al. Health Benefits and Pharmacological Properties of Stigmasterol. Antioxidants. 2022;11(10):1–32. doi: 10.3390/antiox11101912 36290632 PMC9598710

[42] Li X, Zhao M, Tang YR, Wang C, Zhang Z, Peng S. N-[2-(5,5-Dimethyl-1,3-dioxane-2-yl)ethyl]amino acids: Their synthesis, anti-inflammatory evaluation and QSAR analysis. Eur J Med Chem. 2008;43(1):8–18. doi: 10.1016/j.ejmech.2007.03.015 17498849

[43] Mou Y, Meng J, Fu X, Wang X, Tian J, Wang M, et al. Antimicrobial and antioxidant activities and effect of 1-hexadecene addition on palmarumycin C2 and C3 yields in liquid culture of endophytic fungus berkleasmium sp. Dzf12. Molecules. 2013;18(12):15587–99. doi: 10.3390/molecules181215587 24352015 PMC6270283

[44] Abazari M, Ghaffari A, Rashidzadeh H, Badeleh SM, Maleki Y. A Systematic Review on Classification, Identification, and Healing Process of Burn Wound Healing. Int J Low Extrem Wounds. 2022;21(1):18–30. doi: 10.1177/1534734620924857 32524874

[45] Broughton G, Janis JE, Attinger CE. The basic science of wound healing. Plast Reconstr Surg. 2006;117(7 SUPPL.):12–34. doi: 10.1097/01.prs.0000225430.42531.c2 16799372

[46] Landén NX, Li D, Ståhle M. Transition from inflammation to proliferation: a critical step during wound healing. Cell Mol Life Sci. 2016;73(20):3861–85. doi: 10.1007/s00018-016-2268-0 27180275 PMC5021733

[47] Sinno H, Prakash S. Complements and the Wound Healing Cascade: An Updated Review. Plast Surg Int. 2013;2013:1–7. doi: 10.1155/2013/146764 23984063 PMC3741993

[48] Nguyen TT, Mobashery S, Chang M. Roles of Matrix Metalloproteinases in Cutaneous Wound Healing. Wound Heal—New insights into Anc Challenges. 2016

[49] Almohaimeed HM, Al-Zahrani MH, Almuhayawi MS, Algaidi SA, Batawi AH, Baz HA, et al. Accelerating Effect of *Cucurbita pepo* L. Fruit Extract on Excisional Wound Healing in Depressed Rats Is Mediated through Its Anti-Inflammatory and Antioxidant Effects. Nutrients. 2022; 14(16):3336. 36014842 10.3390/nu14163336 PMC9415108

[50] Ayuob N, Hawuit E, M Mohammedsaleh Z, Shaalan D, Mousa Hassn Hawasah M, Abdulrhman Ahmed Basheikh K et al. L. Cucurbita pepo modulates contact dermatitis in depressed rats through downregulation of proinflammatory cytokines and upregulation of antioxidant status. Advances in Dermatology and Allergology/Postępy Dermatologii i Alergologii. 2022;39(2):286–297 doi: 10.5114/ada.2021.103459 35645683 PMC9131963

