# Supplementary material for: Evaluation of anti-inflammatory and wound healing properties of Tinospora cordifolia extract
Source: PLoS One. 2025 Jan 29;20(1):e0317928. doi: 10.1371/journal.pone.0317928 (PMC11778766; doi:10.1371/journal.pone.0317928)
Supplement: S1 File — ANOVA analysis for wound closure. (PDF) [file pone.0317928.s001.pdf]

Supporting Information

Evaluation of Anti-Inflammatory and Wound Healing Properties of *Tinospora cordifolia* Extract

Shohag Chandra Das[1], Subrato Biswas[2], Olin Khan[3], Rupa Akter[2], Md Abul Kalam Azad[1], Sujan Kumar Sarkar[2], Md. Abdul Masum[2*], Sultana Bedoura[1*]

[1]*Department of Dyes and Chemical Engineering, Bangladesh University of Textiles, Dhaka, Bangladesh*

[2]*Department of Anatomy, Histology & Physiology, Sher-e-Bangla Agricultural University, Dhaka, Bangladesh.*

[3]*Department of Wet Process Engineering, Bangladesh University of Textiles, Dhaka, Bangladesh*

SIA Table: Wound diameter (mm) for Control group and Treatment group

Time	Control	Treatment	Control	Treatment	Control	Treatment	Control	Treatment
	(Day 0)		(Day 5)		(Day 10)		(Day 15)	
**Mice 1**	4.5	4.5	3.0	2.0	2.5	1.5	0.5	0.0
**Mice 2**	4.5	4.5	3.0	2.5	3.5	3.0	1.0	0.0
**Mice 3**	4.5	4.5	2.5	2.0	3.0	2.0	0.5	0.0
**Mice 4**	4.5	4.5	3.5	3.0	2.5	1.5	0.5	0.5
**Mice 5**	4.5	4.5	4.0	2.5	3.5	2.0	1.0	0.0

S1B Table: ANOVA analysis for wound closure

Day	Control Group		Treatment Group	
	Mean	Standard Deviation	Mean	Standard Deviation
**Day 0**	4.5	0.000	4.5	0.000
**Day 5**	3.2	0.570	2.4	0.418
**Day 10**	3.0	0.500	2.0	0.612
**Day 15**	0.7	0.274	0.1	0.224

S1C Table: Statistical parameters of wound closure

	F Value	Degrees of Freedom		p-value
		Numerator Degrees	Denominator Degrees	
**Control Group**	105.1228	3.0000	12.0000	< 0.0001
**Treatment Group**	110.0845	3.0000	12.0000	< 0.0001

The p-values for both groups are less than 0.05, indicating significant differences in wound diameters over time within the control and treatment groups.

S1D Table: Post Hoc Tukey Test for Control group

Group 1	Group 2	Mean Difference	p-value	Lower	Upper	Reject Null Hypothesis
**Day 0**	Day 5	1.3	<0.001	1.12	1.48	True
**Day 0**	Day 10	1.5	<0.001	1.32	1.68	True
**Day 0**	Day 15	3.8	<0.001	3.62	3.98	True
**Day 5**	Day 10	0.2	0.758	0.02	0.38	False
**Day 5**	Day 15	2.5	<0.001	2.32	2.68	True
**Day 10**	Day 15	2.7	<0.001	2.52	2.88	True

S1E Table: Post Hoc Tukey Test for Treatment group

Group 1	Group 2	Mean Difference	p-value	Lower	Upper	Reject Null Hypothesis
**Day 0**	Day 5	2.1	<0.001	1.92	2.28	True
**Day 0**	Day 10	2.5	<0.001	2.32	2.68	True
**Day 0**	Day 15	4.4	<0.001	4.22	4.58	True
**Day 5**	Day 10	0.4	0.320	0.22	0.58	False
**Day 5**	Day 15	2.3	<0.001	2.12	2.48	True
**Day 10**	Day 15	1.9	<0.001	1.72	2.08	True

Day 5 vs Day 10: No significant difference in both groups (Control: Mean Difference = 0.2, p-value = 0.758; Treatment: Mean Difference = 0.4, p-value = 0.320)

For the **Control Group**, significant differences are observed between:

- Day 0 and all other days (Day 5, Day 10, Day 15)
- Day 5 and Day 15
- Day 10 and Day 15

For the **Treatment Group**, significant differences are observed between:

- Day 0 and all other days (Day 5, Day 10, Day 15)
- Day 5 and Day 15
- Day 10 and Day 15

SIF: Least Significance Difference (LSD) Test

Code and Calculation

The following Python code was used to calculate the LSD test

```
data = { "Control_Day0": [4.5, 4.5, 4.5, 4.5, 4.5], "Treatment_Day0": [4.5, 4.5, 4.5, 4.5, 4.5],
"Control_Day5": [3, 3, 2.5, 3.5, 4], "Treatment_Day5": [2, 2.5, 2, 3, 2.5], "Control_Day10":
[2.5, 3.5, 3, 2.5, 3.5], "Treatment_Day10": [1.5, 3, 2, 1.5, 2], "Control_Day15": [0.5, 1, 0.5,
0.5, 1], "Treatment_Day15": [0, 0, 0, 0.5, 0] }
df = pd.DataFrame(data)
def calculate_lsd(df, alpha=0.05): results = {} for day in ["Day0", "Day5", "Day10",
"Day15"]: control = df[f"Control_{day}"] treatment = df[f"Treatment_{day}"]
n = len(control)
mean_control = np.mean(control)
mean_treatment = np.mean(treatment)
mse = ((np.var(control, ddof=1) + np.var(treatment, ddof=1)) / 2)
se = np.sqrt(mse * (2 / n))
t_value = t.ppf(1 - alpha / 2, df=(2 * (n - 1)))
lsd = t_value * se mean_diff = np.abs(mean_control - mean_treatment)
significant = mean_diff > lsd
results[day] = {"mean_diff": mean_diff, "LSD": lsd, "significant": significant}
return results
lsd_results = calculate_lsd(df)
lsd_results

'Day0': {'mean_diff': 0.0, 'LSD': 0.0, 'significant': False}, 'Day5': {'mean_diff':
0.8000000000000003, 'LSD': 0.7292225360471934, 'significant': True}, 'Day10':
{'mean_diff': 1.0, 'LSD': 0.8152955806631577, 'significant': True}, 'Day15': {'mean_diff':
0.6, 'LSD': 0.3646112680235967, 'significant': True}}
```

The results of the LSD test were used to determine significant differences between the control and treatment groups at each time point.
